# Efficient Coding and Statistically Optimal Weighting of Covariance among Acoustic Attributes in Novel Sounds

**DOI:** 10.1371/journal.pone.0030845

**Published:** 2012-01-23

**Authors:** Christian E. Stilp, Keith R. Kluender

**Affiliations:** Department of Psychology, University of Wisconsin – Madison, Madison, Wisconsin, United States of America; Rutgers University, United States of America

## Abstract

To the extent that sensorineural systems are efficient, redundancy should be extracted to optimize transmission of information, but perceptual evidence for this has been limited. Stilp and colleagues recently reported efficient coding of robust correlation (*r* = .97) among complex acoustic attributes (attack/decay, spectral shape) in novel sounds. Discrimination of sounds orthogonal to the correlation was initially inferior but later comparable to that of sounds obeying the correlation. These effects were attenuated for less-correlated stimuli (*r* = .54) for reasons that are unclear. Here, statistical properties of correlation among acoustic attributes essential for perceptual organization are investigated. Overall, simple strength of the principal correlation is inadequate to predict listener performance. Initial superiority of discrimination for statistically consistent sound pairs was relatively insensitive to decreased physical acoustic/psychoacoustic range of evidence supporting the correlation, and to more frequent presentations of the same orthogonal test pairs. However, increased range supporting an orthogonal dimension has substantial effects upon perceptual organization. Connectionist simulations and Eigenvalues from closed-form calculations of principal components analysis (PCA) reveal that perceptual organization is near-optimally weighted to shared versus unshared covariance in experienced sound distributions. Implications of reduced perceptual dimensionality for speech perception and plausible neural substrates are discussed.

## Introduction

To the extent that characteristics of a structured world are predictably related, inputs to sensory systems are redundant. It has long been proposed that the role of early sensory processing is to detect, extract, and exploit redundancy in the input [1], [2]. Through processes of evolution and experience, response properties of sensorineural systems should complement statistical regularities of the stimuli to which they are exposed [1]–[8]. These claims of ‘efficient coding’ enjoy a long history in vision research, although direct evidence from perceptual experiments is not abundant [9]. There is physiological evidence that responses of neurons at successive stages of processing become increasingly independent from one another [10], [11], with such demonstrations clearest in the auditory system. For example, Chechik and colleagues [12], [13] report redundancy-reducing transformations of neural responses to bird call stimuli in the ascending auditory pathway of the cat. Auditory cortex responses shared less mutual information (less redundancy, or more independence) compared to neural responses in the inferior colliculus.

Reduction of redundancy has often been inferred from perceptual findings. The most well-known example is the McCollough effect [14], where observers adapt to a contingency between line orientation (horizontal, vertical) and color (red, green), but not to either dimension singly (see [15] for review). Adaptation to complex visual patterns [16]–[18] or to initially arbitrary but thoroughly trained crossmodal contingencies (between luminance and stiffness [19]) provide further examples from which redundancy reduction has been inferred.

One limitation to broad application of efficient coding models is the nearly exclusive investigation of such processes in visual perception. Just as it is true for the optical world, lawful constraints on sound-producing events give rise to natural sounds that are acoustically complex with multiple, redundant attributes. For sounds created by real structures including musical instruments and vocal tracts, changes in different acoustic dimensions cohere in accordance with physical laws governing sound-producing sources. For example, articulatory maneuvers that produce consonant and vowel sounds give rise to multiple acoustic attributes, and changes among these attributes are often correlated [20], [21].

**Figure 1:**
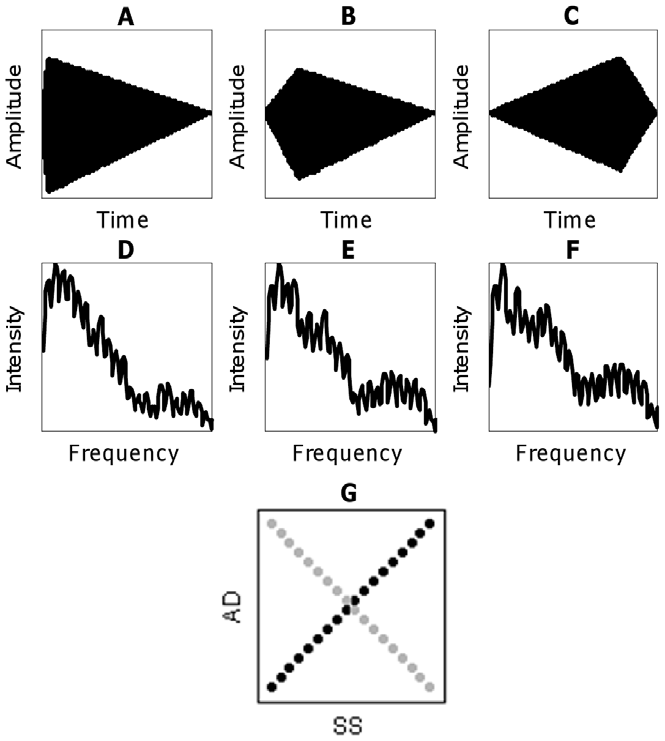
Example stimuli used in the present experiments. The first row shows steps 1 (A; shortest attack/longest decay), 9 (B; intermediate attack/decay) and 18 (C; longest attack/shortest decay) out of 18 in the AD series. The second row shows steps 1 (D; most-French-horn-like), 9 (E; intermediate mixture) and 18 (F; most-tenor-saxophone-like) out of 18 in the SS series, with frequency axes magnified (shown only up to 6 kHz) to emphasize differences in spectral envelopes. The third row shows examples of the two experimental conditions. Black circles depict stimuli that obey a positive correlation between AD and SS (*i.e.*, lie on a main diagonal of the stimulus matrix; Consistent condition). Grey circles depict stimuli that violate that correlation (*i.e.*, lie on the perpendicular diagonal; Orthogonal condition). Examples in 1G depict no overall correlation between AD and SS, but experiments present a high ratio of Consistent∶Orthogonal sounds to introduce correlation among complex acoustic attributes. In counterbalanced conditions, grey sounds support a negative correlation between AD and SS while black sounds directly violate it. Figures 1A and 1D correspond to the black circle in the lower-left corner of 1G, figure 1C to the grey circle in the upper-left corner, and figure 1F to the grey circle in the lower-right corner.

To investigate whether and how auditory perception is sensitive to correlations (redundancy) among acoustic properties, Stilp *et al.* [22] created novel stimuli (heavily edited mixtures of French horn and tenor saxophone samples) that varied along two complex dimensions: attack/decay (AD; Figures 1A–1C) and spectral shape (SS; Figures 1D–1F). Each dimension was independently normed so that all pairs of sounds separated by a fixed number of stimulus steps were approximately equally discriminable. Series were fully crossed to generate a stimulus matrix from which subsets of stimuli were selected to present listeners with either a robust (*r* = ±0.97) or weaker correlation (*r* = ±0.54) between changes in AD and SS. Listeners completed AXB discrimination trials without feedback on stimulus pairs that either respected (Consistent condition) or violated the correlation (Orthogonal, Single-cue conditions). When AD and SS were highly correlated, discriminability of sound pairs obeying the correlation maintained, but became significantly worse for pairs that violate the correlation. This difference in discrimination was evident early in testing, and performance on Orthogonal and Single-cue pairs recovered by the end of the experiment. Conversely, when AD and SS were relatively weakly correlated (*r* = ±0.54), discrimination was equivalent throughout the experiment, suggesting that correlation must be relatively robust to produce differences in discriminability.

Stilp and colleagues tested three unsupervised neural network models, each testing a different hypothesis of how sensorineural systems exploit covariance, to examine how they accounted for listener performance. A Hebbian model [23], [24], in which connection weights adjust in proportion to the correlation between input and output unit activations, predicted reduced discriminability of sounds violating the correlation, but not recovery to baseline levels later in the experiment as observed in listener data. An anti-Hebbian or decorrelation model [16], [25], in which output dimensions become orthogonal via symmetric inhibition between output units proportional to their correlation, predicted superior discrimination of sounds violating the correlation (Orthogonal), contrary to listener performance. Finally, a connectionist simulation of principal components analysis (PCA) [26] predicted the full pattern of results across experiments.

In the PCA network, the first output inhibits inputs to subsequent outputs, thereby removing the principal component from the input pattern and leaving remaining outputs to capture residual covariance. Consistent with listener performance, network outputs initially organized with respect to the principal component (correlation) in the stimulus set, only gradually coming to discriminate or encode remaining variance (orthogonal and single-cue changes).

The statistical purpose of PCA is to linearly transform input data to a new coordinate system for which the greatest amount of variance lies along the first coordinate, or principal component. The second coordinate must be uncorrelated with (orthogonal to) the first and under this restriction, captures the greatest amount of variance not accounted for by the first component. The same restrictions of orthogonality and maximization of variance not yet explained hold for subsequent components. In practice, PCA provides a highly efficient way to represent multidimensional data because derived component dimensions are orthogonal (share no variance), and relatively few components are typically necessary to capture most of the variance in the data. In the present application, there are only two input variables (AD and SS) and thus two components capture all of the variance.

The linear algebraic solution to PCA yields an ordered set of orthogonal components (Eigenvectors) with accompanying weights (Eigenvalues). Each Eigenvalue is proportional to the variance that is accounted for by its associated Eigenvector, and these can be derived from either the covariance matrix or correlation matrix of the input variables. Because covariance among variables is sensitive to units (*e.g.*, degrees Fahrenheit versus Celsius), it is more common to solve for components and weights from a correlation matrix (normalized covariance.) Extending earlier work by Oja [24] and others, Sanger [26] demonstrated that the model employed by Stilp and colleagues [22] finds the Eigenvectors of the input correlation matrix, and is certain to converge to the same solution as closed-form PCA.

To the extent that listener performance can be predicted by PCA, one may infer that redundant attributes are efficiently coded into experience-driven perceptual dimensions at the expense of physical acoustic dimensions. Changes in performance reported by Stilp *et al.* [22] are predicted by correlations between attributes, not attributes AD or SS *per se*. Further, changes in discriminability consequent to nearly-perfect, but not weaker, correlation are functionally sensible. Perceptual representation of the correlation is restricted to cases with sufficiently reliable evidence to limit the perceptual costs (error) of reduced dimensionality.

Why efficient coding was not observed for stimuli with less robust but still notable correlation among attributes (*r* = ±0.54) remains unclear. Relative to the highly-correlated stimulus set presented in Experiment 2 of Stilp *et al.* [22], the less-correlated stimulus set (Experiment 3 in [22]): tested fewer correlated sounds (six versus 18), tested more orthogonal sounds (four versus two), and presented more orthogonal trials overall (three times as many, owing to testing three orthogonal pairs rather than just one). Each manipulation reflects distinct statistical properties that attenuate correlation between AD and SS. As such, each manipulation may contribute differently to perceptual organization and subsequent effects on discrimination, but the perceptual significance of each manipulation is unknown because all were made in concert.

Redundancy between acoustic attributes is attenuated systematically across the following experiments to determine perceptual consequences of different statistical properties of correlations among stimulus attributes that are less than nearly-perfect (Expt. 2, [22]) but greater than that for which no difference in discrimination is observed (Expt. 3, [22]). Separate manipulations of stimulus sets are performed to investigate how strong correlations must be to elicit differential discriminability, and whether different means of attenuating correlation are perceptually equivalent. Predictions made by the PCA neural network are compared to listener performance in each experiment. The model's sensitivity to these manipulations and intermediate correlations is a strong test of its ability to predict listener performance. Finally, how different model predictions, operating on correlation versus covariance matrices, relate to listener performance are explored. Behavioral and computational results support near-optimal weighting of covariance among acoustic attributes.

## Materials and Methods

### 1. Ethics Statement

All experiments were approved by the Education and Social & Behavioral Sciences Institutional Review Board at the University of Wisconsin. Written informed consent was obtained from all participants.

### 2. Listeners

Two hundred undergraduates (40 per experiment, five experiments) from the University of Wisconsin – Madison participated, with no individual participating in multiple experiments. All reported normal hearing, and received course credit in exchange for their participation.

### 3. Stimuli

All stimuli are novel complex sounds described in detail in Stilp *et al.* [22]. Briefly, one waveform period (3.78 ms duration = 264 Hz fundamental frequency) from samples of a French horn and a tenor saxophone [27] was iterated to 500-ms duration. Samples were then edited to vary along one of two complex acoustic dimensions: attack/decay (AD) or spectral shape (SS), dimensions that are in principle relatively independent both perceptually and in early neural encoding [28]. AD was manipulated by varying the amplitude envelope of the stimulus which was set to zero at stimulus onset and offset, with linear ramps from onset to peak and back to offset without any steady state (Figure 1A–C). Attack duration in AD ranged from 20–390 ms in 17 steps (18 stimuli), with decay duration being the remainder of 500 ms (total duration) minus attack duration. SS was manipulated by mixing instrument samples in different proportions, ranging from 0.2 to 0.8 for each instrument and always summing to 1.0 across instruments (*e.g.*, adding 0.4 [French horn]+0.6 [tenor saxophone] to form a new spectral shape). Proportions were derived such that neighboring sounds in the SS series (17 pairs, 18 stimuli total) had equal Euclidean distances between their ERB-scaled magnitude spectra [29] that had been processed through a bank of auditory filters [30] (Figure 1 D–F). Euclidean distance between spectra processed in such a manner has been shown to correspond well with perceptually significant change over time in speech [31]. Specific values for AD and SS series reported above were derived following exhaustive adjustment across hundreds of participants until every pair of sounds separated by three stimulus steps was equally discriminable to every other pair within and across stimulus series (≈65% correct for changes along one dimension, ≈69% for changes along both dimensions; see [22] for details). AD and SS series were fully crossed to generate a 324-sound stimulus matrix. Subsets of this matrix are presented to listeners in all of the following experiments (Figure 1G).

### 4. Experimental Design

#### a. All experiments

**Figure 2:**
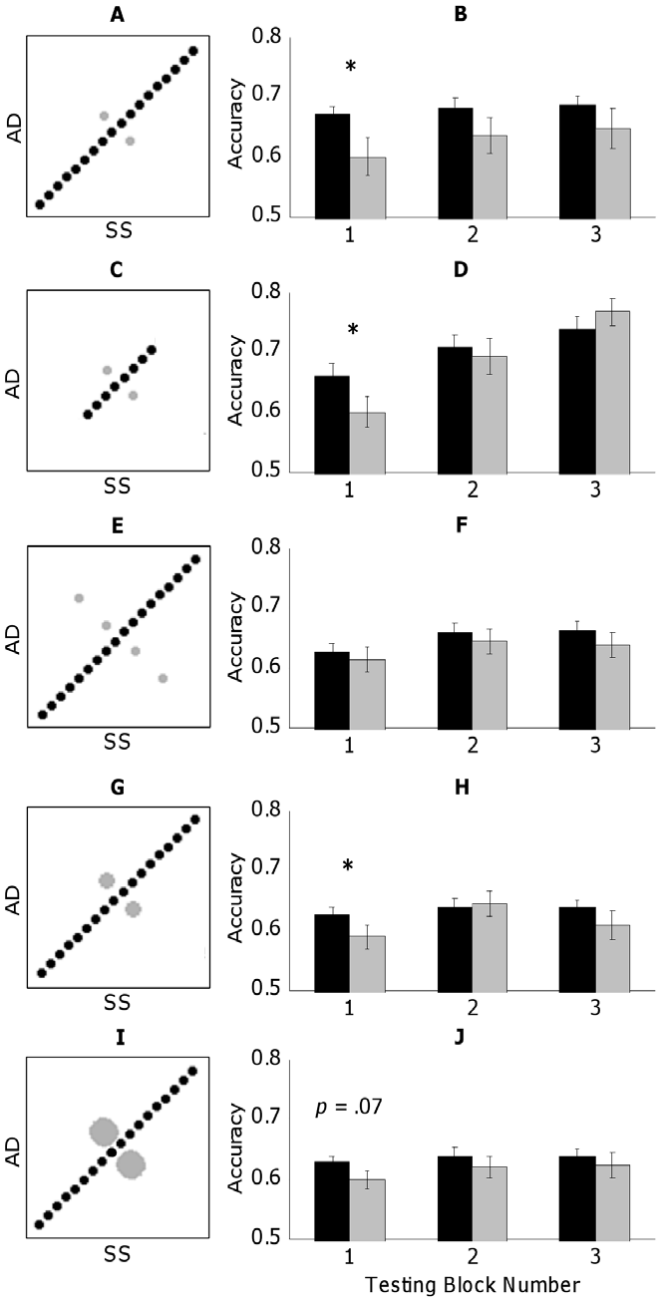
Stimuli and behavioral results for all experiments (black = Consistent condition, grey = Orthogonal condition). Stimulus representations follow Figure 1G. While only positive correlations are shown, experiments were counterbalanced between positive and negative correlations. All behavioral results depict proportion correct discrimination on the ordinate and testing block number on the abscissa. Stimuli (A) and results (B) for Experiment 1 (base design; *r* = ±0.98). Stimuli (C) and results (D) for Experiment 2 (truncation of evidence supporting the correlation; *r* = ±0.81). Stimuli (E) and results (F) for Experiment 3 (expansion of evidence supporting the orthogonal dimension; *r* = ±0.83). Stimuli (G) and results (H) for Experiment 4 (threefold increase in sampling Orthogonal stimuli; *r* = ±0.95). Stimuli (I) and results (J) for Experiment 5 (tenfold increase in sampling Orthogonal stimuli; *r* = ±0.83). * indicates significant difference (*p*<.05) as assessed by paired-sample two-tailed *t*-tests.

All experiments employ designs similar to those reported by Stilp *et al.* [22] with one notable change. While Stilp *et al.* [22] also assessed discrimination of sounds varying along AD or SS with the other dimension fixed (Single-cue stimuli), those trials are eliminated here so that all performance comparisons are made between experimental conditions in which both acoustic cues change. Stimuli belong to one of two conditions: sounds that lay along the main diagonal of the stimulus matrix, conforming to the robust correlation between AD and SS (Consistent condition), or sounds that lay along the perpendicular diagonal that bisects the matrix, directly violating this correlation (Orthogonal condition; see Figure 1G). Each experiment is counterbalanced such that twenty participants discriminated stimuli with a positive correlation between AD and SS, and twenty discriminated stimuli with a negative correlation (*i.e.*, 90° rotation of stimuli depicted in Figure 2). Thus, one group's Orthogonal stimuli serve as Consistent stimuli to the other group and *vice versa*. Sounds in the Consistent condition are arranged into pairs each separated by three stimulus steps, and likewise for Orthogonal sounds. Each stimulus pair was presented in all possible AXB triads (AAB, ABB, BAA, BBA) with 250-ms ISIs.

Correlation coefficients were calculated for each stimulus set using nominal values from 1 to 18 to represent AD and SS values. Without any sounds along the perpendicular (orthogonal) diagonal, the correlation between AD and SS would equal 1. Across experiments, different stimuli presented in the Orthogonal condition attenuate this correlation to varying degrees.

#### b. Experiment 1

Experiment 1 serves as a replication of Experiment 2 in Stilp *et al.* [22], but without any Single-cue stimuli. Successful replication permits Experiment 1 to serve as the base design for Experiments 2 through 5, in which the correlation among acoustic attributes is systematically violated to evaluate the perceptual significance of different statistical characteristics of the stimulus set and whether they promote or hinder efficient coding. The Consistent condition was comprised of all 18 sounds (15 pairs) along the main diagonal of the stimulus matrix, and the Orthogonal condition was comprised of 2 sounds (one pair) along the perpendicular diagonal, resulting in a nearly-perfect correlation between AD and SS (*r* = ±0.98; Figure 2A).

#### c. Experiment 2

Experiment 2 tests the degree to which differences in discriminability (Consistent versus Orthogonal) are sensitive to the physical acoustic/psychoacoustic range of exemplars supporting the correlation (*i.e.*, the diagonal bisecting the stimulus matrix) relative to the variability supporting the orthogonal dimension. By reducing the extent of evidence supporting the correlation, listeners may more quickly discover variability not explained by the correlation, resulting in comparable discrimination across conditions throughout the experiment. Two sounds on the Orthogonal diagonal are arranged into one stimulus pair as before, but the range over which AD and SS covary is truncated from 18 to eight sounds (15 pairs to five), reducing the correlation between AD and SS (*r* = ±0.81; Figure 2C).

#### d. Experiment 3

Experiment 3 examines whether perception is sensitive to the range of variance orthogonal to the correlation. Stimulus sets tested in Experiments 1 and 2 included only two Orthogonal sounds, both located very close to the correlated diagonal in the stimulus matrix. However, the less-correlated stimulus set tested in Experiment 3 of Stilp *et al.* [22] included both these two proximal Orthogonal sounds and two more extreme sounds, presenting a wider range of evidence violating the correlation. Presentation of Orthogonal sounds increasingly distinct from the correlation (*i.e.*, located further away from the diagonal in the stimulus matrix) may contribute to listeners discovering this variance more quickly, reducing or even eliminating significant differences in discrimination early in testing. In a review of visual adaptation studies, Kohn [32] notes that adaptation effects are directly affected by the similarity between adaptor and test item. By decreasing similarity between conditions through presentation of more extreme Orthogonal sounds, listeners' adaptation to the contingency between AD and SS (*i.e.*, differences in discriminability depending on whether trials respect or violate the correlation) should reduce in magnitude, duration, or both. Stimuli in Experiment 3 consist of 18 sounds on the correlated diagonal (15 pairs) and the same four sounds on the orthogonal diagonal (3 pairs) as presented in Experiment 3 of [22] (*r* = ±0.83; Figure 2E).

#### e. Experiment 4

By adding more extreme Orthogonal sounds to the stimulus set, Experiment 3 tests three Orthogonal pairs rather than the one pair tested in Experiments 1 and 2, thus conflating the extent of Orthogonal evidence with increased probability of Orthogonal pairs. Experiment 4 unconfounds these factors, examining changes in discriminability as a function of the simple probability of Orthogonal test trials. The lone Orthogonal pair presented in Experiments 1 and 2 was tested three times as often as each of the 15 Consistent pairs, producing the same ratio of Consistent-to-Orthogonal test trials as in Experiment 3. Increasing the probability of the Orthogonal pair threefold only slightly reduces the correlation between AD and SS (*r* = ±0.95; Figure 2G).

#### f. Experiment 5

The possibility exists that any significant differences in discriminability in Experiment 4 may be attributable to the robustness of correlation (*r* = ±0.95) rather than probability of Orthogonal test trials (presented three times as often as any Consistent trial). Experiment 5 presents a stronger test by increasing the frequency of Orthogonal test trials until the strength of correlation is equated to that of Experiment 3 (*r* = ±0.83). This was accomplished by presenting the lone Orthogonal pair 10 times as often as any given Consistent pair (15 total; Figure 2I).

### 4. Procedure

Sounds were upsampled to 48828 Hz, D/A converted (Tucker-Davis Technology RP2.1), amplified (TDT HB4), and presented diotically over circumaural headphones (Beyer Dynamic DT-150) at 72 dB SPL. Following acquisition of informed consent, between one and three individuals participated concurrently in single-subject soundproof booths. Each participant heard trials in a different randomized order. Trials were presented twice in each of three blocks in Experiments 1 and 3, and were presented three times per block in Experiment 2 in order to produce an experimental session of comparable overall duration. In Experiments 4 and 5, the Orthogonal pair is deliberately oversampled. No feedback was provided. Listeners were given the opportunity to take a short break between testing blocks. Owing to the varying numbers of trials presented (E1: 128 trials/block, 384 trials total; E2: 72 trials/block, 216 trials total; E3: 144 trials/block, 432 trials total; E4: 144 trials/block, 432 trials total; E5: 200 trials/block, 600 trials total), experiments had different durations (E1: 25 min; E2: 15 min; E3: 30 min; E4: 30 min; E5: 40 min).

### 5. Computational Modeling

#### a. Correlation-based model

**Figure 3:**
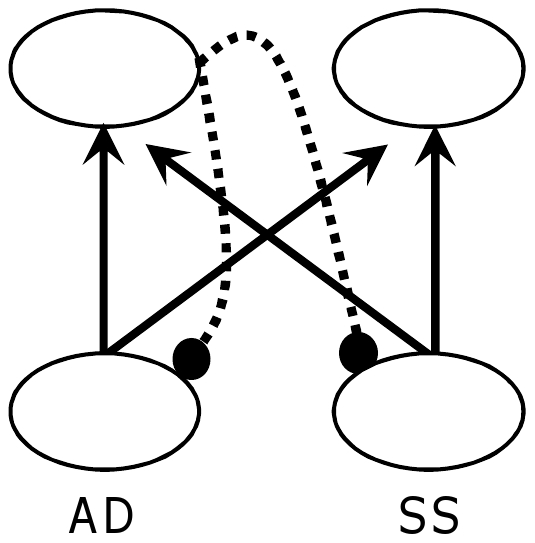
PCA network architecture. Two input units (one corresponding to AD, one to SS) are fully connected to two output units via feed-forward excitatory weights (solid arrows) without any hidden layer or bias. The first output unit projects inhibitory weights (dashed lines) back to the inputs, effectively removing the principal component from the inputs and leaving the second output to encode remaining (orthogonal) covariance. Euclidean distances among output patterns were calculated after each epoch.

The same unsupervised PCA network model [26] employed by Stilp *et al.* [22] was used. This model discovers Eigenvectors based on the correlation matrix of the inputs. The present experiments demonstrate this aspect of the standard model (versus calculating Eigenvectors from the covariance matrix of the inputs) to be a perceptually important one, and its success predicting results of Stilp *et al.* [22] makes it an appropriate starting point. The model featured two input units (one corresponding to AD, the other to SS) which were fully connected in a feed-forward manner to two output units with no hidden layer and no bias (Figure 3). Inhibitory connections projected from the first output back to input units at a fixed value of 1. Output activations and subsequent effects on input states were implemented serially: the first output unit was activated; its activation was “subtracted out” of the input values; then, the second output unit was activated. Feed-forward weights were trained using standard Hebbian learning, resulting in the first output unit representing the principal component of the inputs while the second output captured residual (orthogonal) covariance. Importantly, while closed-form (linear algebraic) PCA calculates Eigenvectors and corresponding components simultaneously, the model calculates these elements iteratively. The rate at which the model learns the second component (as reflected by decreased Euclidean distances between Orthogonal stimuli compared to Consistent stimuli before returning to baseline) is of key interest in the comparison to listener data.

The model was initialized with weights (2-by-2 identity matrix) that ensured output patterns initially mirrored input patterns. Weights ultimately converge to Eigenvectors of the input correlation matrix, organized in decreasing Eigenvalue order. Simulations were comprised of continuous testing with a small learning rate. The model was trained with analogs of each stimulus set, with 18 steps of AD and SS normalized and coded as values −8.5 through 8.5. Euclidean distances calculated between output patterns (*i.e.*, representations of stimulus pairs) after each epoch provide a model analog of perceptual discriminability.

**Figure 4:**
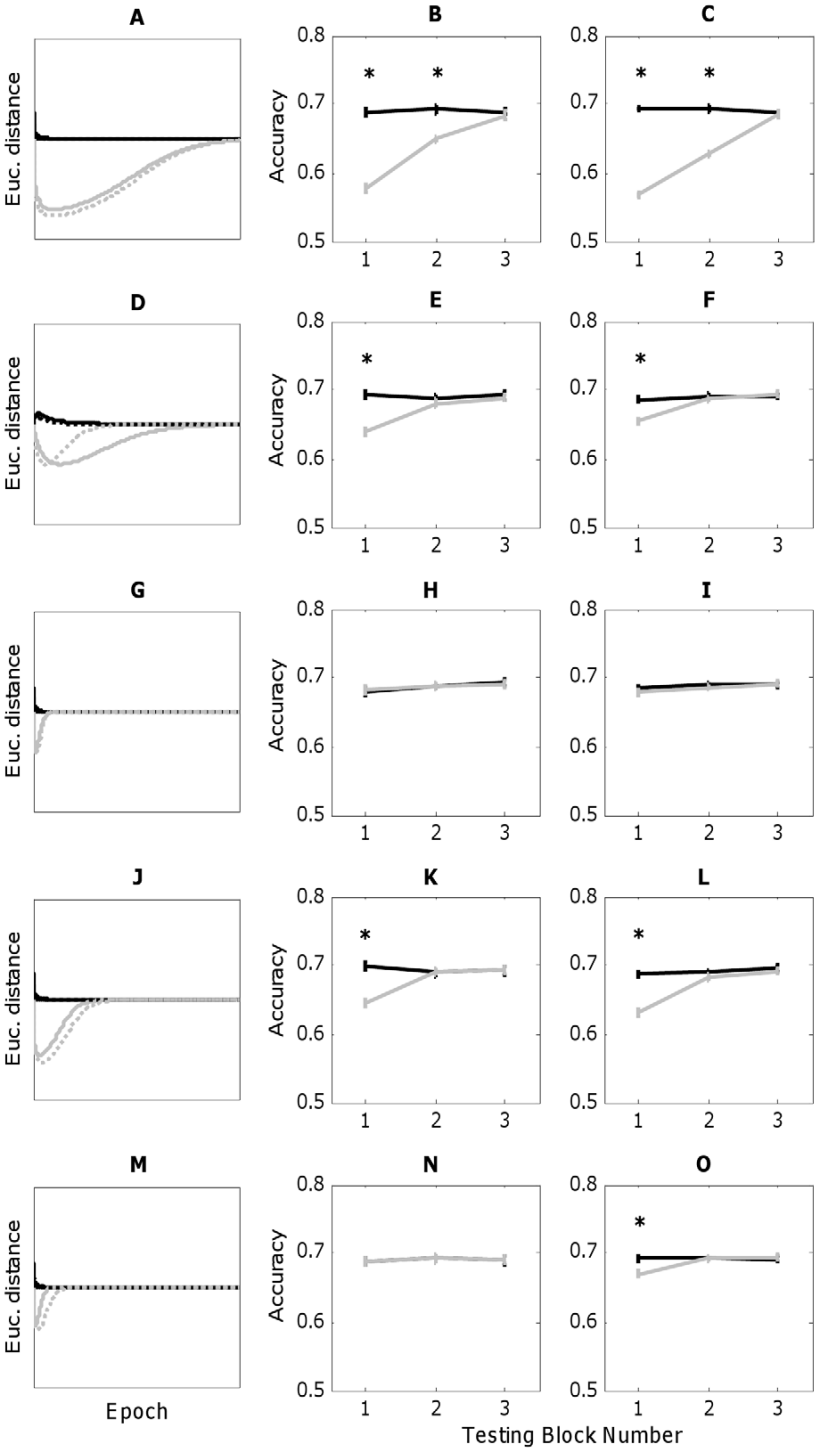
PCA network simulations (left column) and choice model performance (center, right columns) for all experiments (black = Consistent condition, grey = Orthogonal condition). The first row corresponds to Experiment 1, the second row to Experiment 2, *etc.* In PCA simulations (A, D, G, J, M), Euclidean distance between test stimuli is on the ordinate and simulation epoch on the abscissa. Solid lines portray predictions made by the correlation-based model, while (often highly overlapping) dashed lines portray predictions of the covariance-based model. Choice model performance (center, right columns) plots proportion correct discrimination on the ordinate and testing block number on the abscissa. Choice model performance based on the correlation-based PCA model is shown in the center column (B, E, H, K, N), and performance based on the covariance-based PCA model is shown in the right column (C, F, I, L, O). Choice model patterns of performance for both correlation and covariance are identical for Experiments 1–4. However, the correlation model fails to predict listeners' superior discrimination of statistically consistent sound pairs (O) early in Experiment 5 (N) while the covariance-based model successfully predicts this performance.

Simulations were conducted for a standard duration of 500 epochs for ease of visualization and comparison across experiments. Simulation of all experiments achieved convergence (no further changes in weights) following this duration except for Experiment 1, which reached convergence after 600 epochs. The reasons for portraying the first 500 epochs of this simulation are twofold. First, the first 500 epochs are plotted to better illustrate changes in Euclidean distances early in the simulation, which are of principal interest as discriminability is predicted to be equivalent across conditions later in the experiment. Second, Euclidean distances and weights associated with the second Eigenvector (Orthogonal stimuli) were within 2% of their final values at 500 epochs, so the model makes qualitatively the same prediction at both points in the simulation – that Consistent and Orthogonal stimuli should be equally discriminable. Simulation results are presented in the left (solid lines) column of Figure 4.

#### b. Covariance-based model

The present effort reveals an important limitation of Sanger's [26] PCA model. By calculating Eigenvectors of the inputs based on their correlation matrix, the model will produce identical predictions for dissimilar stimulus sets that have the same correlation coefficients. Correlation is the normalized version of covariance, calculated as the covariance between two variables divided by the product of their standard deviations. Thus, one stimulus set with greater covariance between variables and greater standard deviations may produce the same correlation coefficient as a different stimulus set with lesser covariance between variables with smaller standard deviations.

For example, consider the case for the model predictions of Experiments 3 and 5 (Figure 4). Relatively few stimuli in Experiment 3 violate the correlation (four Orthogonal sounds), contributing to covariance of 20.93. However, inclusion of more extreme Orthogonal sounds with greater distances away from the main (correlated) diagonal results in a higher standard deviation of 5.02 for AD and for SS. Conversely, extreme oversampling of the lone Orthogonal pair in Experiment 5 reduces the covariance between AD and SS (11.88). However, the proximity of these stimuli to the diagonal decreases stimulus variability, as reflected by smaller standard deviations (3.78). Despite stark differences in the Orthogonal information in each stimulus set, each experiment maintains the same correlation between AD and SS (20.93/5.02^2^ = 11.88/3.78^2^ = 0.83). Despite different covariances and covariance matrices, these stimulus sets possess the same correlation coefficients and correlation matrices, and the correlation-based PCA model makes identical predictions for both despite any potential differences in listener performance across experiments.

It is common to conduct PCA using the correlation matrix in order to normalize out effects of scaling. However, acoustic dimensions AD and SS were thoroughly piloted by Stilp *et al.* [22] to assure that steps along each dimension were equally discriminable absent experimental effects of redundancy among attributes. Thus, stimuli are designed to be psychophysically normalized. Using the correlation-based model imposes additional normalization on stimuli that have already been perceptually normalized. Subsequently, covariance among attributes may better reflect perceptual processes for the present stimuli. Models of Hebbian-type learning based on covariance have been used to model long-term depression of synaptic strength in the hippocampus [33]–[35]. Further, a covariance-based model is capable of making different predictions for stimuli with the same correlation matrices but different covariance matrices. Thus, a PCA model that operates on the covariance matrix of the inputs may be a more appropriate means of predicting listener performance.

Sanger's [26] PCA model was modified to operate on the covariance matrix of the inputs in the following manner. Equation 1 depicts Sanger's original algorithm ([26], p. 465):
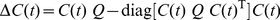
(1)where *C* represents the weight (Eigenvector) matrix, *Q* represents the correlation matrix of the inputs, diag indicates elements on the main diagonal of the matrix, and ^T^ denotes matrix transposition. In the present application, weight changes are calculated at each epoch of the simulation, so (*t*) is implied and thus omitted for simplicity. Through the mathematical proof that the Generalized Hebbian Algorithm produces Eigenvectors of the input correlation matrix ordered by decreasing Eigenvalue, Sanger ([26], p. 462) expressed Equation 1 in terms of each row of the weight matrix as follows:
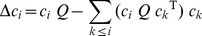
(2)where *c_i_* represents the *i*th row of the weight matrix, and *c_k_* represents the *k*th row of the weight matrix, which spans from 1 to *i*. The reader will note that *c_i_* ^T^ represents a row in Sanger's notation and *c_i_* represents a column; these notations are reversed here for ease of reading so that row elements are assumed and transpositions denote columns. Expanding Equation 2 into a separate equation for each row of the weight matrix yields Equations 3.1 (principal component) and 3.2 (second component):
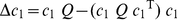
(3.1)

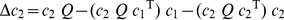
(3.2)Equations use multiplicative normalization (subtraction of (*c*_1_ *Q c*_1_^T^)*c*_1_ in Equation 3.1 and (*c*_2_ *Q c*_2_^T^)*c*_2_ in Equation 3.2) so the sum of squared weights remains constant; otherwise weights grow without bound. Subtraction of the term (*c*_2_ *Q c*_1_^T^)*c*_1_ in Equation 3.2 removes the principal component from calculations so that weight changes are derived solely from unshared covariance. These equations were revised by substituting the covariance matrix of the inputs, represented by *E*, for the correlation matrix *Q*, as shown in Equations 4.1 and 4.2:
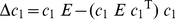
(4.1)

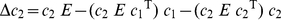
(4.2)Weight changes are scaled by a small learning rate (*η* = 0.01). To compare simulations of a given experiment using covariance and correlation versions of the PCA model, covariance-based simulations continued until reaching a specified criterion: matching the ratio between Orthogonal and Consistent Euclidean distances at the 500^th^ epoch of the simulation of Experiment 1 using the correlation-based model (ratio = 0.9848). This criterion was selected to make depictions of correlation- and covariance-based model simulations comparable, as all begin and end with the same relationships (ratios) between Orthogonal and Consistent Euclidean distances. This criterion was met at the 1015^th^ epoch of simulating Experiment 1 using the covariance-based model, thus all covariance-based model simulations span 1015 epochs. Simulation results are presented in the left column of Figure 4 (dashed lines) superimposed atop results for the correlation-matrix-based model (solid lines) for comparison.

#### c. Comparison to listener performance

**Figure 5:**
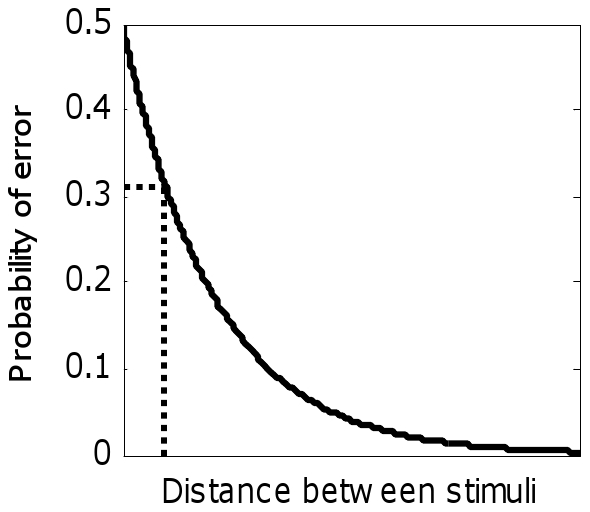
The choice model of Xu *et al.* [**36**], where the probability of error in a two-alternative forced-choice (AXB) task decreases exponentially with increasing distance between stimuli (solid line). Dashed lines correspond to error probability of 0.31, or baseline discriminability between experimental stimuli absent effects of correlation among attributes [22], and the corresponding inter-stimulus distance.

Neural network model predictions were quantitatively tested using the general metric learning procedure of Xu, Zhu, and Rogers [36], which translates computed distances between stimuli into probability of a correct response in a discrimination task. This ‘choice model’ assumes that stimulus confusions (errors in a two-alternative forced-choice [AXB] task) decrease as a function of distance between two stimuli, such that increasing distances result in improved discriminability (Figure 5). This function is expressed in Equation 5:
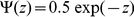
(5)with *z* corresponding to distance between stimuli and Ψ the probability of an incorrect response on a discrimination trial. While error probability can decay in either exponential or Gaussian manners with increasing distance, the former is employed here (see [36] for discussion). Baseline performance, or discriminability of experimental stimuli absent effects of correlation, corresponds to an error rate of 0.31 (69% correct discrimination [22]). Distances along the abscissa of Figure 5 were scaled so that Euclidean distances at the beginning and convergence of the PCA model simulation corresponded to this baseline error rate.

The output of PCA model simulations (respective Euclidean distances between Consistent and Orthogonal stimuli measured at each epoch) served as inputs to the choice model. At each epoch, for Consistent and Orthogonal conditions, Euclidean distance between stimuli was converted into the corresponding error rate (Ψ). A random number uniformly distributed between 0 and 1 was then generated (*n*). Each ‘trial’ was scored as correct if *n*>Ψ and incorrect if *n*≤Ψ. Similar to human data, ‘trials’ were divided into three blocks of equal size, and error rates were averaged across all ‘trials’ within a block. This process was repeated 40 times with different random seeds to simulate data from 40 human participants. Results were averaged across these 40 runs of the choice model, and means and standard errors for proportion of trials correctly discriminated (calculated as 1 minus error rate, matching portrayal of listener data) are presented in Figure 4. Simulation results are assessed using paired-sample *t*-tests, following analysis of listener performance. Choice model simulations were conducted separately for distances calculated by the correlation-matrix-based and covariance-matrix-based versions of the PCA model.

## Results

### 1. Listener performance

Behavioral results from all experiments are presented in the right column of Figure 2, with discrimination accuracy (proportion correct) on the ordinate and testing block number on the abscissa. Given that Orthogonal discriminability is predicted to recover by the end of the experiment, omnibus analysis of variance (ANOVA) tests are likely to result in Type II error. Consequently, to retain sensitivity to differences in discriminability across conditions at different phases of the experiment, results are analyzed using planned-comparison paired-sample *t*-tests.

#### a. Experiment 1

Discrimination of Consistent pairs in the first block of testing (mean = 0.67, s.e. = .01) was significantly better than discrimination of Orthogonal pairs (mean = 0.60, s.e. = .03) (*t*_39_ = 2.36, *p*<.025, Cohen's *d* = 0.44; Figure 2B). While discrimination accuracy of Consistent pairs was numerically greater than that of Orthogonal pairs in the second (mean of 0.68 versus 0.63) and third testing blocks (0.69 versus 0.65), *t*-tests did not reach statistical significance (second block: *t*_39_ = 1.58, *p* = .12; third block: *t*_39_ = 1.27, *p* = .21). This pattern of results replicates Experiment 2 of Stilp *et al.* [22]; discrimination of Orthogonal test pairs is initially inferior to that of Consistent test pairs supporting a robust correlation, and performance recovers through further testing so that discrimination across conditions is comparable by the final testing block. It bears mention that in their Expt. 2 (*r* = 0.97), Stilp *et al.* [22] report superior discrimination of Consistent sound pairs relative to Orthogonal sound pairs in the first as well as second testing block. Relative to that experimental design, Expt. 1 in the present report removes Single-cue stimuli while maintaining 18 Consistent sounds and 2 Orthogonal sounds yielding nearly the same correlation (*r* = 0.98). In the present experiment, Consistent discrimination was significantly more accurate than Orthogonal discrimination in the first testing block (*p*<.025) with only a trend toward significance in the second testing block (*p* = 0.12). It is unclear why the full pattern of significance was not fully replicated despite highly similar stimuli and correlation coefficients. Independent-samples *t*-test indicates that the difference in Consistent and Orthogonal discrimination in the second testing block did not significantly differ across experiments (*t*_78_ = 0.73, *p* = 0.47), suggesting patterns of results are not fundamentally different from one another. Results indicate that both the correlated and orthogonal dimensions appear to become weighted proportional to the amount of variance accounted for by each dimension.

#### b. Experiment 2

Discrimination of Consistent pairs in the first block of testing (mean = 0.66, s.e. = .02) was again significantly better than discrimination of Orthogonal pairs (mean = 0.60, s.e. = .03) (*t*_39_ = 2.71, *p*<.01, Cohen's *d* = 0.43; Figure 2D). Despite restricting the range of acoustic evidence supporting the correlation, this early difference in discrimination persisted. Experiment 2 also reveals that correlation among stimulus attributes need not be nearly perfect (*r*≥0.97) for efficient coding to occur. Discrimination did not significantly differ in either the second (Consistent mean = 0.71, s.e. = .02, Orthogonal mean = 0.69, s.e. = .03; *t*_39_ = 0.67, *n.s.*) or third block (Consistent mean = 0.74, s.e. = .02, Orthogonal mean = 0.77, s.e. = .02; *t*_39_ = 1.27, *n.s.*).

Unlike previous experiments, discrimination in both conditions improved markedly across testing blocks. Owing to the inability to separate learning (improvement throughout the experiment) from effects of the correlation between AD and SS on Orthogonal discriminability (initially inferior but later comparable to that of Consistent sound pairs), performance was assessed through paired-sample *t*-tests contrasting early versus late (*i.e.*, first versus third testing block) discrimination of Consistent pairs, which are predicted to remain equally discriminable throughout the experiment. Consistent discrimination significantly improved from the first to third block of Experiment 2 (*t*_39_ = 4.39, *p*<.0001, Cohen's *d* = 0.60), but this learning effect was not consistent across experiments. Participants in Experiment 3 exhibited a significant but more modest learning effect for Consistent trials (*t*_39_ = 3.23, *p*<.01, Cohen's *d* = 0.35), but no significant differences were observed in Experiments 1, 4, or 5 (all *t*≤1.21, *n.s.*, Cohen's *d*<0.18). The magnitude of the learning effect in Experiment 2 may be due to one or both of the following factors. First, reducing variability in AD and SS cues by truncating the correlation may facilitate discrimination over time. Second, listeners in Experiment 2 were presented more repetitions of stimulus pairs in a given block (12) than in other experiments (8) in the effort to make overall number of trials comparable. Nevertheless, the principal finding is superior discrimination of Consistent pairs relative to Orthogonal pairs early in testing.

#### c. Experiment 3

Unlike previous experiments, discrimination was comparable across Consistent (mean = 0.63, s.e. = .01) and Orthogonal conditions (mean = 0.61, s.e. = .02) in the first testing block (*t*_39_ = 0.75, *n.s.*, Cohen's *d* = 0.12; Figure 2F). By testing more extreme Orthogonal test pairs (*i.e.*, less similar to Consistent pairs), differences in discrimination observed in previous experiments were extinguished. Roughly equivalent discrimination persisted throughout the experiment (Block 2: Consistent mean = 0.66, s.e. = .02, Orthogonal mean = 0.64, s.e. = .02 [*t*_39_ = 0.86, *n.s.*]; Block 3: Consistent mean = 0.66, s.e. = .02, Orthogonal mean = 0.64, s.e. = .02 [*t*_39_ = 1.54, *n.s.*]). This demonstrates that efficient coding of correlated acoustic attributes is sensitive to the range of physical acoustic/psychoacoustic evidence inconsistent with the primary correlation and consistent with a second orthogonal dimension. Results also demonstrate that simple strength of the primary correlation is insufficient to attenuate discriminability of orthogonal stimulus differences, as all stimulus pairs presented in Experiment 3 (*r* = ±0.83) were relatively equally discriminable, but pairs presented in Experiment 2 (*r* = ±0.81) produced significant differences in early performance. The explanatory power of simple strength of correlation between acoustic attributes, absent consideration of both the quantity and quality (range) of evidence that is inconsistent with the correlation, is challenged by these results.

#### d. Experiment 4

Despite a three-fold increase in presentations, discrimination of the Orthogonal pair (mean = 0.59, s.e. = .02) was still significantly worse than that of Consistent pairs (mean = 0.63, s.e. = .01) in the first testing block (*t*_39_ = 2.06, *p*<.05, Cohen's *d* = 0.37; Figure 2H). This negligible effect of probability sheds light on the results of Experiment 3, that efficient coding was likely extinguished due to increased range of acoustic evidence supporting orthogonal variability and not the concurrent increase in Orthogonal test trials. Similar to previous experiments, performance across conditions was equivalent in the second (Consistent mean = 0.64, s.e. = .02, Orthogonal mean = 0.64, s.e. = .02 [*t*_39_ = 0.36, *n.s.*]) and third testing blocks (Consistent mean = 0.64, s.e. = .01, Orthogonal mean = 0.61, s.e. = .02 [*t*_39_ = 1.58, *n.s.*]).

#### e. Experiment 5

Even with ten-fold oversampling, discrimination of the Orthogonal pair (mean = 0.60, s.e. = .02) was modestly worse than that of Consistent pairs (mean = 0.63, s.e. = .01) in the first testing block (*t*_39_ = 1.87, *p* = .07, Cohen's *d* = 0.36; Figure 2J). It bears note that paired-sample *t*-tests used in all analyses are two-tailed. One could use a one-tailed *t*-test based on the prediction that discrimination of Consistent pairs will be greater than that of Orthogonal pairs, in which case the difference would be statistically significant (one-tailed *p*<.05). However, performance in the first block does not significantly differ in Experiment 5 versus Experiment 3 as indicated by independent samples *t*-tests on orthogonal discrimination performance (*t*_78_ = 0.63, *n.s.*) and differences between Consistent and Orthogonal discrimination (*t*_78_ = 0.71, *n.s.*). Perhaps surprisingly, testing the Orthogonal sound pair ten times as often as any Consistent sound pair failed to produce practice effects sufficient to promote Orthogonal discrimination exceeding Consistent discrimination (second block: Consistent mean = 0.64, s.e. = .02, Orthogonal mean = 0.62, s.e. = .02 [*t*_39_ = 0.69, *n.s.*]; third block: Consistent mean = 0.64, s.e. = .01, Orthogonal mean = 0.62, s.e. = .02 [*t*_39_ = 0.54, *n.s.*]). Thus, the conservative conclusion one can draw from this marginal effect is that manipulation of Orthogonal stimulus probability has little effect on listener discrimination.

### 2. Model predictions

#### a. Experiment 1

Predictions from the PCA models are presented in the first column of Figure 4, with Euclidean distance between Consistent (black) versus Orthogonal (grey) stimulus pairs on the ordinate and training epoch on the abscissa. Simulation timecourses for correlation-matrix-based (solid lines) and covariance-matrix-based (dashed lines) models are scaled to share comparable abscissas. Similar to [22], the PCA model quickly discovered the principal component (the Consistent dimension) and distances between Orthogonal pairs initially decreased considerably (Figure 4A). With further exposure to the stimulus set, the PCA model gradually captured the modest variance not explained by the first component, progressively increasing distances between Orthogonal pairs until reaching original relative values by the end of the simulation. Thus, the PCA model initially captures only variability along the principal component in the two-dimensional stimulus space at the expense of the orthogonal component, incrementally coming to capture remaining variance, matching the pattern observed in listener performance. Predictions from the correlation-based (solid lines) and covariance-based (dashed lines) versions of the PCA model were nearly identical, with a slightly larger initial decrease in Orthogonal distances predicted by the covariance model.

Simulation results using the choice model are depicted in the middle (correlation) and right (covariance) columns of Figure 4, with percent correct discrimination along the ordinate and testing block number along the abscissa. Predictions across 40 simulations exhibited markedly less variability than listener data, but patterns of results remain excellent fits to human performance. Both correlation and covariance models predicted significantly poorer discrimination of Orthogonal stimuli in the first block of testing (correlation model [Figure 4B]: Consistent: mean = 0.69, s.e. = .006; Orthogonal: mean = 0.58, s.e. = .006, *t*_39_ = 14.92, *p*<1e-17, Cohen's *d* = 3.15; covariance model [Figure 4C]: Consistent: mean = 0.69, s.e. = .004; Orthogonal: mean = 0.57, s.e. = .004, *t*_39_ = 21.50, *p*<4e-23, Cohen's *d* = 5.09). Marked improvement in Orthogonal discrimination was evident in the second block, but this was still inferior to Consistent discrimination (correlation model: Consistent: mean = 0.69, s.e. = .007; Orthogonal: mean = 0.65, s.e. = .005, *t*_39_ = 5.23, *p*<6e-6, Cohen's *d* = 1.19; covariance model: Consistent: mean = 0.69, s.e. = .004; Orthogonal: mean = 0.63, s.e. = .004, *t*_39_ = 10.12, *p*<2e-12, Cohen's *d* = 2.38). Finally, Consistent and Orthogonal stimuli were relatively equally discriminable in the third block (correlation model: Consistent: mean = 0.69, s.e. = .005; Orthogonal: mean = 0.68, s.e. = .006, *t*_39_ = 0.62, *n.s.*; covariance model: Consistent: mean = 0.69, s.e. = .004; Orthogonal: mean = 0.68, s.e. = .004, *t*_39_ = 0.39, *n.s.*).

#### b. Experiment 2

The initial decrease in distance between Orthogonal stimuli is smaller and recovery to baseline distances sooner than that observed for Experiment 1 (Figure 4D). These outcomes are anticipated given simulation of a more weakly correlated stimulus set (*r* = ±0.81). Simulations by Stilp *et al.* [22] and Experiment 1 suggest that principal and second components become weighted in proportion to the amount of covariance captured by each dimension, and model predictions for Experiment 2 reveal more weight being attributed to the second (Orthogonal) dimension as it captures relatively more unshared covariance here than in other, more highly-correlated stimulus sets. Both correlation-based and covariance-based models predict significantly poorer Orthogonal discrimination in the first testing block, but models make different predictions regarding the rate of recovery to baseline distances between stimuli. The correlation-based model predicts a more extended recovery, which contributes to a larger predicted effect size in the first block (Consistent: mean = 0.69, s.e. = .006; Orthogonal: mean = 0.64, s.e. = .006, *t*_39_ = 5.65, *p*<2e-6, Cohen's *d* = 1.40; Figure 4E) than that predicted by the covariance-based model (Consistent: mean = 0.69, s.e. = .004; Orthogonal: mean = 0.65, s.e. = .005, *t*_39_ = 4.95, *p*<2e-5, Cohen's *d* = 1.12; Figure 4F), which predicts more rapid recovery to baseline distances. Nevertheless, both models correctly predict significantly poorer Orthogonal discrimination in the first testing block, and comparable discrimination in the second (correlation model: Consistent: mean = 0.69, s.e. = .004; Orthogonal: mean = 0.68, s.e. = .007, *t*_39_ = 1.12, *n.s.*; covariance model: Consistent: mean = 0.69, s.e. = .004; Orthogonal: mean = 0.69, s.e. = .004, *t*_39_ = 0.62, *n.s.*) and third testing blocks (correlation model: Consistent: mean = 0.69, s.e. = .006; Orthogonal: mean = 0.69, s.e. = .005, *t*_39_ = 0.38, *n.s.*; covariance model: Consistent: mean = 0.69, s.e. = .004; Orthogonal: mean = 0.69, s.e. = .004, *t*_39_ = 0.48, *n.s.*), matching listener performance. Finally, neither version of the PCA model predicts overall improved performance later in the simulation (*i.e.*, Euclidean distances in both conditions increasing over time) as observed in listener performance, suggesting insensitivity to some practice effects.

#### c. Experiment 3

Both versions of the PCA model predict a shallow and very short-lived decrease in Orthogonal distances, with the vast majority of the simulation predicting equal discriminability across conditions (Figure 4G). Virtually identical simulation results both predict comparable performance across conditions in the first (correlation model [Figure 4H]: Consistent: mean = 0.68, s.e. = .006; Orthogonal: mean = 0.68, s.e. = .005, *t*_39_ = 0.26, *n.s.*; covariance model [Figure 4I]: Consistent: mean = 0.68, s.e. = .004; Orthogonal: mean = 0.68, s.e. = .004, *t*_39_ = 0.75, *n.s.*), second (correlation model: Consistent: mean = 0.69, s.e. = .005; Orthogonal: mean = 0.69, s.e. = .006, *t*_39_ = 0.08, *n.s.*; covariance model: Consistent: mean = 0.69, s.e. = .003; Orthogonal: mean = 0.69, s.e. = .004, *t*_39_ = 0.60, *n.s.*), and third testing blocks (correlation model: Consistent: mean = 0.69, s.e. = .005; Orthogonal: mean = 0.69, s.e. = .006, *t*_39_ = 0.25, *n.s.*; covariance model: Consistent: mean = 0.69, s.e. = .004; Orthogonal: mean = 0.69, s.e. = .004, *t*_39_ = 0.26, *n.s.*). These predictions mirror listener performance, and support the idea that both listeners and the model quickly exploited covariance in more extreme Orthogonal stimuli to discover the second component and facilitate Orthogonal discrimination.

#### d. Experiment 4

Both versions of the PCA model predict a sizable initial decrease in Orthogonal distances before later recovery to original relative distances (Figure 4J). These predictions resemble those of Experiment 1, where the early difference in discrimination was both predicted and behaviorally observed, in contrast to those of Experiment 3, where largely equal discrimination throughout was both predicted and observed. Recovery to original relative distances for Orthogonal stimuli occurred much more quickly in Experiment 4 than Experiment 1, revealing some sensitivity to the fact that Orthogonal stimuli were sampled more frequently. Further, the covariance model predictions displayed a slightly larger magnitude of initial decrease in Orthogonal distances and slightly longer recovery to baseline distances than that observed for the correlation model, resulting in a slightly larger effect size in the first testing block (correlation model (Figure 4K): Consistent: mean = 0.70, s.e. = .005; Orthogonal: mean = 0.64, s.e. = .005, *t*_39_ = 6.94, *p*<3e-8, Cohen's *d* = 1.65; covariance model (Figure 4L): Consistent: mean = 0.69, s.e. = .004; Orthogonal: mean = 0.63, s.e. = .005, *t*_39_ = 7.85, *p*<2e-9, Cohen's *d* = 1.89). Both versions of the model predicted equal discriminability in the second (correlation model: Consistent: mean = 0.69, s.e. = .006; Orthogonal: mean = 0.69, s.e. = .005, *t*_39_ = 0.14, *n.s.*; covariance model: Consistent: mean = 0.69, s.e. = .004; Orthogonal: mean = 0.68, s.e. = .005, *t*_39_ = 1.20, *n.s.*) and third testing blocks (correlation model: Consistent: mean = 0.69, s.e. = .006; Orthogonal: mean = 0.69, s.e. = .005, *t*_39_ = 0.12, *n.s.*; covariance model: Consistent: mean = 0.70, s.e. = .005; Orthogonal: mean = 0.69, s.e. = .004, *t*_39_ = 0.62, *n.s.*).

#### e. Experiment 5

The correlation-based PCA model predicts a shallow and very short-lived decrease in Orthogonal distances, with all but the first few epochs of the simulation predicting equal discriminability across conditions (Figure 4M). These predictions are identical to those made for Experiment 3, such that equal discriminability of Consistent and Orthogonal stimuli is predicted in all blocks of testing (Block 1: Consistent: mean = 0.69, s.e. = .005; Orthogonal: mean = 0.69, s.e. = .006, *t*_39_ = 0.13, *n.s.*; Block 2: Consistent: mean = 0.69, s.e. = .005; Orthogonal: mean = 0.69, s.e. = .007, *t*_39_ = 0.06, *n.s.*; Block 3: Consistent: mean = 0.69, s.e. = .006; Orthogonal: mean = 0.69, s.e. = .006, *t*_39_ = 0.09, *n.s.*; Figure 4N).

Similar to Experiment 4, the covariance-based PCA model predicts a slightly larger magnitude of initial decrease in Orthogonal distances and slightly longer recovery to baseline distances than that observed for the correlation model (Figure 4M). These differ from other model predictions in two significant ways. First, similar to listeners and unlike the correlation model, the covariance model predicts inferior discrimination of Orthogonal stimuli in the first testing block of Experiment 5 (Consistent: mean = 0.69, s.e. = .004; Orthogonal: mean = 0.67, s.e. = .004, *t*_39_ = 4.02, *p*<.0005, Cohen's *d* = 0.87; Figure 4O). Second, the covariance model displays sensitivity to (and thus makes different predictions for) stimuli with the same correlation matrix but different covariance matrices (*i.e.*, stimuli presented in Experiments 3 and 5). An independent-samples *t*-test confirms that the predicted difference in Consistent and Orthogonal discrimination in the first testing block of Experiment 5 (mean difference = .023) is significantly larger than the difference observed in the first block of Experiment 3 (mean difference = .005; *t*_78_ = 2.11, *p*<.05). Predictions made by the correlation model for the first block of Experiment 3 versus Experiment 5 did not differ (independent-samples *t*-test on mean differences: *t*_78_ = 0.28, *n.s.*). These results demonstrate that while the PCA model based on the correlation matrix of the inputs [26] is useful for predicting discriminability of some stimulus sets, the covariance-based PCA model is a better predictor of listener performance overall. Finally, the covariance model predicted comparable performance across conditions for remaining test blocks (Block 2: Consistent: mean = 0.69, s.e. = .004; Orthogonal: mean = 0.69, s.e. = .004, *t*_39_ = 0.08, *n.s.*; Block 3: Consistent: mean = 0.69, s.e. = .004; Orthogonal: mean = 0.69, s.e. = .004, *t*_39_ = 0.42, *n.s.*).

#### f. Across all experiments

The predictive power of covariance-based PCA is further demonstrated through closed-form linear algebraic solutions in Table 1. Table 1 orders stimulus sets from Experiments 1–5 to reflect performance differences in discriminability of Consistent versus Orthogonal sound pairs in the first testing block as measured by effect size (rightmost column). Eigenvalues calculated from the correlation matrix versus covariance matrix of stimulus set before the simulation are also provided. The success with which listeners discriminate Orthogonal pairs is well predicted by the second Eigenvalue calculated from the covariance matrix reflecting true psychoacoustic distances: as the second Eigenvalue increases, greater perceptual weighting is reflected in improved listener performance on Orthogonal trials and subsequently decreased effect sizes early in the experiment (*r* = −0.95, *p*<.025). This relationship with performance is not observed for the second Eigenvalue of correlation matrices, the first Eigenvalue of correlation or covariance matrices, or simple strength of the principal correlation. The relationship between the second Eigenvalue of the covariance matrix and effect size is similarly robust if calculated on model representations of the inputs after the first one-third of the simulation (akin to the first testing block for listeners; *r* = −0.94, *p*<.025). No other metric calculated after one-third of the simulation reliably predicts effect sizes for the first block of testing. While some caution is warranted in generalizing this relationship given that the second Eigenvalue can be increased by multiple manipulations (removal of Consistent sounds, addition of more extreme Orthogonal sounds, oversampling of Orthogonal sounds), it does provide promising extensions of the present work in optimal weighting of statistically derived dimensions in complex sounds.

**Table 1:** Correlation coefficients (*r*), first and second Eigenvalues (λ_1_, λ_2_), covariance between AD and SS (σ_AD,SS_), and effect sizes (Consistent versus Orthogonal discrimination in the first testing block, as measured by Cohen's *d*) for each experiment.

		Correlation Model			Covariance Model		Effect
	*r*	λ_1_	λ_2_	σ_AD,SS_	λ_1_	λ_2_	Size
Exp. 1	0.98	1.98	0.02	25.26	51.00	**0.47**	0.44
Exp. 2	0.81	1.81	0.19	4.17	9.33	**1.00**	0.43
Exp. 4	0.95	1.95	0.05	20.48	42.13	**1.17**	0.37
Exp. 5	0.83	1.83	0.17	11.88	26.19	**2.43**	0.36
Exp. 3	0.83	1.83	0.17	20.93	46.14	**4.29**	0.12

Correlation Model indicates Eigenvalues calculated from the correlation matrix of the stimulus sets before the simulation, while Covariance Model indicates Eigenvalues calculated from the input covariance matrix before simulations. The order of experiments is intentionally transposed to highlight the robust negative correlation between the second Eigenvalue of the covariance matrix of the experimental stimuli with listener performance.

## Discussion

The present results replicate and extend reports by Stilp *et al.* [22] of rapid efficient coding of redundancy among acoustic dimensions in novel complex sounds. Three manipulations, each of which attenuates correlation among attributes, were tested separately to examine the perceptual significance of each. Overall, simple strength of the primary correlation (principal component) is inadequate to predict listener performance. Initial superiority of discrimination for statistically consistent sound pairs was relatively insensitive to truncation of evidence supporting the correlation (Experiment 2) and to increases in the frequency of Orthogonal test trials (Experiments 4, 5). However, increased evidence of an orthogonal dimension provided by greater acoustic/psychoacoustic range (Experiment 3) proved highly salient, resulting in equivalent discrimination performance throughout the experiment.

Patterns of performance cannot be explained by independent weighting of acoustic dimensions (AD, SS), as changes in discriminability can only be attributed to the correlation or covariance orthogonal to it. This perceptual adherence to derived statistical structure, and not physical acoustic dimensions *per se*, is not without precedent. There is good evidence that auditory cortical representations decreasingly correspond to physical stimulus dimensions [37]–[39]. Wang [39] refers to this as “non-isomorphic” transformations of the input. Examples of non-isomorphic stimulus representations in auditory cortex include encoding spectral shape across varying absolute frequencies [38], gross representation of rapid change in click trains with short inter-click intervals versus phase-locking to trains with slower inter-click intervals [40], [41], and encoding pitch versus individual frequency components [42], [43]. Such non-isomorphic transformations may be similar to the loss of acoustic dimensions (AD, SS) seen here, as more efficient dimensions better capture perceptual performance. Results are in agreement with Stilp and Kluender [44], who report efficient coding of redundant acoustic dimensions in the face of unrelated variability in a third acoustic feature.

Optimal combination and weighting of individual stimulus dimensions has received considerable attention in vision research. Models of Bayesian inference and ideal perceptual performance have been shown to effectively capture aspects of perception of objects [45], [46], edges [47], movement [48], and slant or orientation [49]–[52]. These ideal observer models have been extended to perceptual combination of sensory cues from different modalities, such as integrating visual and auditory cues to location [53], visual and motor cues to performing certain actions [54]–[57], and visual and haptic cues to height [58], shape [59], and even thoroughly trained arbitrary associations such as one between luminance and stiffness [19].

Three important points distinguish these earlier studies from the present findings in auditory perception. First, such studies often must address inherent weights or biases ascribed to each cue. For example, visual information is habitually weighted more heavily than auditory or haptic information. Here, acoustic dimensions AD and SS were adjusted through extensive control studies to be equally available perceptually, so *a priori* perceptual weights are equated. Second, many cue weighting studies examine performance as a function of relative noisiness (relative σ) of respective cues. Sensibly, when multiple cues are available but one is or becomes more noisy (larger σ), perceptual weights are greater for less noisy cues that better inform behavior. Optimal cue combination occurs when one cue (typically the one weighted more heavily absent experimental manipulation) is made noisier and perceptual weights shift toward a less noisy source of information (*e.g.*, making the visual signal noisier and observing increased weight attributed to haptic information [58]). Cues AD and SS share equal psychoacoustic variability as measured by JNDs. Third and most importantly, these examples from vision or multimodal research demonstrate optimal weighting of individual physical stimulus dimensions. The present findings indicate optimal weighting of derived dimensions that capture statistical relationships between attributes. This likely suggests a more sophisticated level of processing than that observed for reports of combination or integration of individual physical stimulus cues.

Behavioral results were consistently predicted by the PCA network model [26]. Perceptual processes first capture the principal component of variation in the two-dimensional stimulus space at the expense of the orthogonal component [22]. From listener performance and models, it appears that both principal and second components become weighted proportional to the amount of variance accounted for by each. In the stimulus sets tested here, this entailed relatively modest weights on the second component, corresponding to initially reduced discriminability. Following further exposure to the stimulus set, variance not explained by the principal correlation is detected and exploited, improving discrimination of Orthogonal sound pairs back to baseline levels. Only when evidence for the orthogonal dimension was increased through greater covariance not shared with the principal component (Experiment 3) was sufficient weight attributed to the second component, extinguishing early differences in discriminability. Otherwise, given that correlations tested here were attenuated in different manners, simulations primarily varied in how the initial decrease in Euclidean distance between Orthogonal stimuli gets smaller and/or recovery to baseline distances occurs sooner.

One shortcoming of Sanger's [26] network model is that it assumes the correlation matrix of the inputs. PCA can operate over either a correlation or covariance matrix, and there are reasons to prefer a covariance matrix for psychoacoustically-normed experimental materials employed here. The predictive power of the PCA model [26] was improved when modified to operate on the covariance matrix of the input rather than the correlation matrix. The modified model provided predictions that better fit listener performance. Further, Eigenvalues from covariance- but not correlation-based PCA analyses closely reflect listener performance (Table 1). Greater Eigenvalues on the second component (orthogonal to the main correlation) predicted better discrimination of orthogonal variation. At least for these stimuli, covariance among acoustic attributes appears to be a better estimate of perceptual performance than correlation, but given markedly different ways to manipulate covariance captured by a particular component in PCA (stimulus addition/deletion, over/undersampling, *etc.*), further studies are required to better understand this relationship.

The particular PCA model investigated here [26] is certainly oversimplified and is unlikely to precisely reflect neural learning mechanisms. Dimensions of AD and SS are almost certainly encoded across a large number of neurons and not the localist representation tested here. A more serious challenge is to identify neurally plausible mechanisms for instantiating PCA-like performance. Conceivably, circuitry of auditory cortical and association areas may provide the required connectivities. Precortical processes might also be implicated, given that PCA has proven practical for depicting correlations across neurons in the vibrissal sensory area of rat thalamus [60]. Lower subcortical auditory nuclei are also candidates given that, relative to the visual system, much more processing (more synapses and hence greater neural recoding) occurs within the brainstem before cortex [37]. Identification of neural substrates supporting perceptual changes demonstrated here and by Stilp and colleagues [22] would facilitate development of more authentic computational models.

The present experiments have investigated how listeners adapt to strong covariance structure coupled with varying types of orthogonal variation. This form of structure is particularly amenable to decomposition via PCA, but other models are better suited for a broader array of cases such as those presented by statistical distributions for some speech sounds (*e.g.* distributions of vowels in formant (F_1_-F_2_-F_3_) space are not orthogonal). For extraction of independent dimensions that are not necessarily orthogonal, techniques such as linear independent component analysis (ICA), which efficiently encodes structure into latent components that minimize mutual information (redundancy) between outputs (*e.g.*, [61]), may provide a better statistical analog to perceptual organization.

The present results could provide insights into models of perceptual organization for complex sounds such as speech. While the novel sounds tested here only varied along two complex dimensions, patterns of covariance naturally scale to high-dimensional feature spaces. In complex natural stimuli such as speech, multiple forms of stimulus attribute redundancy exist concurrently and successively [20], [21], [62]–[65]. To the extent that patterns of covariance among acoustic attributes in natural sounds are efficiently coded, the present results may inform how the auditory system exploits different patterns of redundancy to learn and distinguish different speech sounds.

While some have suggested the importance of correlations among stimulus attributes are central to perceptual organization for speech [22], [63], [66]–[68], it has been more common to emphasize 1^st^-order statistics (*e.g.*, probability density) as a means to characterize distributions of speech sounds [69]–[73] or cues [74]–[77]. In experiments that oversampled the Orthogonal sound pair (Experiments 4 and 5), manipulations of probability density had little to no effect on patterns of performance. At least in this particular paradigm, higher-order redundancy (covariance) was more perceptually salient than lower-order redundancy (probability density). Future research that explores relative influences of these different types of statistical structure will inform models of perceptual organization and categorization of speech.

Covariance among complex acoustic attributes in novel stimuli is exploited quickly and automatically in the present experiments. Perception only later comes to encode residual variability in ways that reflect optimal statistical weighting of covariance not accounted for by the principal component of the stimuli. Results illuminate stimulus characteristics that support coding of stimulus redundancy that is rapid, unsupervised, efficient, and statistically optimal.
